# Is education level, as a proxy for socio-economic position, related to device-measured and self-reported sedentary behavior in European older adults? A cross-sectional study from the SITLESS project

**DOI:** 10.3389/fpubh.2023.1296821

**Published:** 2023-12-19

**Authors:** Beatriz Rodriguez Roca, Mark A. Tully, Oriol Sansano-Nadal, Paolo Caserotti, Laura Coll-Planas, Marta Roqué, Jan Brønd, Nicole E. Blackburn, Jason J. Wilson, Dietrich Rothenbacher, Emma McIntosh, Manuela Deidda, Elena Andrade-Gómez, Maria Giné-Garriga

**Affiliations:** ^1^Department of Physiatry and Nursing, Faculty of Health Sciences, University of Zaragoza, Zaragoza, Spain; ^2^Institute of Mental Health Sciences, School of Health Sciences, Ulster University, Newtownabbey, United Kingdom; ^3^Department of Physical Activity and Sport Sciences, Faculty of Psychology, Education and Sport Sciences (FPCEE) Blanquerna, Ramon Llull University, Barcelona, Spain; ^4^School of Health and Sport Sciences (EUSES), Rovira i Virgili University, Amposta, Spain; ^5^Department of Sports Science and Clinical Biomechanics, Center for Active and Healthy Ageing (CAHA), Syddansk Universitet, Odense, Denmark; ^6^Fundació Salut I Envelliment (Foundation on Health and Ageing) – UAB, Universitat Autònoma de Barcelona, Barcelona, Spain; ^7^Iberoamerican Cochrane Centre - Biomedical Research Institute Sant Pau (IIB Sant Pau), CIBER Epidemiología y Salud Pública (CIBERESP), Barcelona, Spain; ^8^Institute of Nursing and Health Research, School of Health Sciences, Ulster University, Newtownabbey, United Kingdom; ^9^Sport and Exercise Sciences Research Institute, School of Sport, Ulster University, Newtownabbey, United Kingdom; ^10^Institute of Epidemiology and Medical Biometry, Ulm University, Ulm, Germany; ^11^Health Economics and Health Technology Assessment (HEHTA), Institute of Health and Wellbeing (IHW), University of Glasgow, Glasgow, United Kingdom; ^12^Department of Nursing, Faculty of Health Sciences, University of La Rioja, Logroño, Spain; ^13^Department of Physical Therapy, Faculty of Health Sciences (FCS) Blanquerna, Ramon Llull University, Barcelona, Spain

**Keywords:** older adults, sedentary behavior, education level, mentally active sedentary behavior, mentally passive sedentary behavior older adult, sedentary, mentally passive sedentary behavior, accelerometer

## Abstract

**Background:**

Sedentary behavior (SB) is a determinant of health in older adult people. Educational level is a primary driver of health disparities and is demonstrated to be a reliable measure of socioeconomic position. We aimed to examine the associations between educational level and self-reported along with device-measured SB in older adults living in Europe and the association of mentally active and passive SB domains with the educational level and gender in these associations.

**Methods:**

The design is cross-sectional. One thousand three hundred and sixty participants aged 65 and over (75.3±6.3 years old, 61.8% women) participated. Inclusion criteria were scored with the Short Physical Performance Battery. Variables that describe the sample were assessed with an interview, and device-measured SB was assessed with an accelerometer. SB was assessed with the Sedentary Behavior Questionnaire and an accelerometer. Multiple linear regression models were used to study the association between the level of education and SB.

**Results:**

Participants self-reported an average of 7.82 (SD: 3.02) daily waking hours of SB during weekend days, and the average of device-measured SB was 11.39 (1.23) h. Total mentally active SB (weekdays and weekends) was associated with the education level (*p* < 0.000). Participants were more sedentary during the week than during weekends, regardless of level of education (*p* < 0.000). Education level was significantly associated with self-reported mean hours per day in 46SB (*p* = 0.000; *R*=0.026; 95%CI).

**Conclusion:**

Low education level in older adults is associated with self-reported SB but not with objective SB measures.

## 1 Introduction

Sedentary behavior (SB) has emerged as a new risk factor for health (1) and is defined as a behavior with low energy expenditure (1.5 metabolic equivalents [METs]), while in a sitting or reclining posture during waking hours, distinct from the simple absence of physical activity (PA) (2). SB has been positively associated with obesity and chronic diseases such as cardiovascular disease (3), decreased cognitive function (4), risk of depression (5), diabetes (4), some types of cancer and mortality (6), and increased vulnerability to falls and fractures (7). Importantly, replacing SB with the same amount of moderate-to-vigorous physical activity (MVPA) may contribute to better quality of life in older adults (8). Older adults are the most sedentary of all age groups (9, 10). SB tends to be more frequent after retirement from full-time work (11), when the older adult is alone at home (12) as well as after lunch and evening meals. Recent studies concluded that older adults spend an average of 9.4 h/day in SB according to objective measures (13), which represents between 65 and 79% of their awake time (14).

Several accelerometer devices have proven to be reliable and valid (e.g., ActiGraph (15, 16), activPAL (17), and Axivity (18)). Self-reported questionnaires, such as the Sedentary Behavior Questionnaire (SBQ), have also been used to subjectively measure SB in large samples due to their low administration cost, ease of administration, and ability to distinguish between different domains where SB takes place (19). Nevertheless, data from self-reported instruments should be taken with caution due to their weak-to-moderate correlation with device-measured SB in the older adult population (20, 21). However, self-reported questionnaires can collect the domains where SB takes place during a usual day.

Time spent in different domains of SB (such as screen time or transport) is likely to differ between older adults. Most studies have focused on only one SB domain or on overall SB time (22), making it difficult to assess the correlates of various domains (10). Recent studies have classified SB into two categories according to its cognitive stimulation: mentally active and passive SB. Mentally active SB, such as reading a book or playing a musical instrument, could be protective and promote healthy cognition, whereas mentally passive SB such as watching television has been associated with increased levels of depressive symptoms (23). For example, Hallgren et al. (24) concluded that not all sedentary activities have equal negative impacts on health; mentally passive SB was more associated with declines in physical function and mental health outcomes than mentally active SB (24).

Educational level seems to be associated with SB in adults according to European-based studies (25–27). The highest education level achieved has shown to be a good indicator of socioeconomic position (SEP) (26) and has been described as a fundamental cause of inequalities on health (28). It is considered a protective factor for the health of the older adult population and establishes the basis for a healthy lifestyle, and it is an indicator of stability throughout life (29). The highest prevalence of unhealthy behaviors happens among the population of lower SEP (30–32).

There is evidence that older persons from socioeconomically poor backgrounds are more likely to be sedentary (10). These factors include a complex interplay between social, physical, and cultural settings and health-related characteristics. On the one hand, prior research from middle-class and high-income nations (*n* = 101) found an inverse relationship between SB and a high SEP that was linked to a higher education level (33). Additionally, it was associated with higher occupation-based SB (34) and higher PA levels in subjects. Conversely, older persons with lower SEP and lower education levels are likely to engage in SB activities such as watching television (35) more frequently than older adults with higher SEPs and higher education levels. On the other hand, some research revealed the contrary, showing that the greater the adults’ level of education, the greater their overall SB time (36).

To further prioritize future investments, it is necessary to identify and understand modifiable factors such as SB and its domains (mentally active and passive), as well as the extent to which it is related to education levels, in order to develop appropriate policies and effective interventions to reduce SB time and modify its patterns.

The activities carried out in each age group are associated with gender. In general, in the older adult population, men are more sedentary than women, analyzing them from the objective and subjective perspective (37, 38). Additionally, disruptions to sedentary behaviors also vary by gender. For example, women perform more SB interruptions than men. In women, they take place in the morning and, in men, throughout the day (37, 39).

Given that the SB levels in the older adult population are increasing, and the current literature on the socioeconomic determinants of SB in European older adults is still limited (40), we aimed to examine the associations between the educational level and self-reported as well as device-measured SB in a large sample of community-dwelling older adults living in four European countries. Furthermore, given that there is not enough bibliography that analyzes the relationship between gender and mentally active or passive sedentary behaviors, we have decided that our secondary aims were to assess the association of several SB domains including mentally active and passive SB with older adults’ educational level and to assess the role of gender in the associations.

## 2 Materials and methods

### 2.1 Design and study population

The SITLESS study was a cross-sectional study. It was a multicenter pragmatic three-armed parallel randomized controlled trial. Community-dwelling older adults aged 65 years or older, with a score on the Short Physical Performance Battery (SPPB) of four or above (41), who were insufficiently active and/or reported high levels of SB (42), were recruited in study centers in Denmark, Spain, United Kingdom, and Germany according to their existing primary prevention pathways. The present cross-sectional study uses data from the baseline assessments. A total of 1,360 community-dwelling older adults (75.3 ± 6.3 years old, 61.8% women) were analyzed at baseline. The Ethics and Research Committee of each intervention site approved the study design. Participation was voluntary, and all participants signed informed consent before the start of the study.

### 2.2 Procedure

In each of the health centers participating in the study, potential patients will be asked about their participation. The members of the research team will report on the objectives of the study. In addition, there will be a strategy to attract future participants through audiovisual means. Once patients begin to show interest in participating, the scientific team will be in charge of verifying that they meet all the inclusion criteria to be able to comment on the study. The study protocol can be found elsewhere (43).

### 2.3 Measures

Personal information regarding age, gender, country, number of current medical conditions, and body mass index (BMI) was collected by means of a structured interview in the study centers. Weight and height were objectively measured by a trained researcher using a TANITA BC 420 and a SECA 213 portable stadiometer, respectively, to derive the participants’ BMI.

The highest education level achieved was collected at the interview with the following answer options: (a) I cannot read or write; (b) primary education (primary education, preindustrial learning, elementary school); (c) secondary education (high school, professional learning, industrial official, industrial expert, commercial sector); (d)university; (e) unwilling to answer.

According to Hallgren et al., we operationalized mentally active SB as the first five domains of the Sedentary Behavior Questionnaire, which included computer work, paperwork, reading, playing an instrument, and artwork. We operationalized mentally passive SB as the other four domains: television, listening to music, using the phone, and driving in a car (24). Participants self-reported the number of hours spent sitting on a weekday and on a weekend day in different domains using the Sedentary Behavior Questionnaire (SBQ).

All participants wore an ActiGraph wGT3X-BT triaxial accelerometer (ActiGraph, LLC, Pensacola, FL) on their dominant hip during waking hours for 7 consecutive days, removing it only for water-based activities such as bathing or swimming and to sleep during the night. Participants recorded the wear time in an activity diary. The devices were initialized to collect data at 30 Hz. To be included in the analysis, an accelerometer record needed to contain at least 4 valid days (including at least 1 weekend day), with a valid day defined as containing at least 600 min (10 h/d) of wear time as in previous studies (44). Non-wear time was defined using a two-window system: a 90-min window for checking for consecutive zero counts and another 30-min upstream and window for checking for more than 2 min of non-zero counts (45). Due to some participants wearing the ActiGraph during nighttime sleeping, a maximum daily wear time was set at 19 h using a pragmatic choice based on participants’ diaries and sleep time duration recommendations for older adults (46). For relevant participants, a log diary was used to determine daily wear time when awake. SB was defined as <100 counts per minute (CPM) (47) on the vertical axis. Values were normalized to the total wear time. Raw accelerometer data were analyzed using ActiLife v6.13.3 software with the normal filter and summarized into 10-s epochs, as have been recommended for the estimation of SB in clinical older adult populations (48). Total daily SB time including time spent sitting and lying was used for data analysis.

### 2.4 Statistical analyses

Baseline cross-sectional characteristics were presented descriptively as mean and standard deviation (SD) for continuous variables and percentage for categorical variables. The normality of the variables was explored with the Kolmogorov–Smirnov test, and owing to its positive result, the non-parametric Kruskal–Wallis test was utilized. To compare the qualitative variables, the *X*^2^ test was used.

Analysis of variance (Kruskal–Wallis test) was used to examine differences across levels of education and continuous variables: age, number of medical conditions, self-reported hours per week of SB during weekdays and weekend days, and accelerometry derived daily hours of SB.

The associations between educational level and total daily SB time, time in each SB domain of the SBQ, and overall, mentally active and passive SB, were analyzed through multiple linear regression for each outcome with covariates: age, gender, country, number of medical conditions, and category of BMI (obese when BMI was ≥30 kg/m^2^, overweight when BMI was 25–29.9 kg/m^2^, normal weight when BMI was 18.5–24.9 kg/m^2^, underweight when BMI was less than 18.4 kg/m^2^). The results represented for the whole week, during week, and weekend days separately. Models with device-measured SB were also adjusted for accelerometer wear time. The results were reported as unstandardized regression coefficients with 95% confidence intervals. All analyses were conducted with IBM SPSS Statistics 26.0. The significance level was set at *p* < 0.05.

## 3 Results

Of the overall SITLESS participants (*n* = 1,360), 137 participants were excluded as they did not meet the pre-specified ActiGraph wear time criteria or did not complete the self-reported questionnaire and interview. A final subsample of 1,223 participants (75.20 ± 6.19 years old, 61.4% women) returned valid accelerometer data and completed the SBQ and the interview. N = 283 reported having primary education (23.14%, 77.06 ± 5.95 years old), 662 secondary education (54.13%, 75.16 ± 6.10 years old), and 278 university education (22.73%, 73.38 ± 6.11). In the primary education level group, 41.9% were obese whereas in the secondary and university education level groups. The overall self-reported mean hours per day in SB were 7.83 ± 3.04 on weekdays and 7.54 ± 2.91 at weekends (Table 1).

**Table 1:** Characteristics of the overall sample according to the education level and divided by gender.

	Primary education				Secondary education				Univesity	*p*
	Male (*n* = 66)	Female (*n* = 217)	Total (*n* = 283)	Male (*n* = 266)	Female (*n* = 396)	Total (*n* = 662)	Male (*n* = 140)	Female (*n* = 138)	Total (*n* = 278)	
Age (years), mean (SD)	77.56 (5.58)	76.91 (6.06)	77.06 (5.95)	75.99 (6.33)	74.61 (5.88)	75.16 (6.10)	73.29 (6.12)	73.47 (6.13)	73.38 (6.11)	0.000^a^
SBQ hours/day, mean (SD)	6.89 (2.39)	7.42 (3.2)	7.30 (3.04)	7.84 (2.71)	8.08 (2.92)	7.99 (2.84)	7.36 (2.21)	7.93 (2.94)	7.64 (2.61)	<0.001^a^
SBQ WD hours/day, mean (SD)	6.94 (2.59)	7.47 (3.37)	7.35 (3.21)	7.92 (2.86)	8.14 (3.11)	8.05 (3.01)	7.47 (2.46)	8.07 (3.21)	7.77 (2.86)	
SBQ WE hours/day, mean (SD)	6.74 (2.36)	7.27 (3.28)	7.15 (3.09)	7.63 (2.81)	7.92 (2.94)	7.80 (2.89)	7.06 (2.36)	7.57 (3.00)	7.31 (2.70)	
Accelerometry:										<0.000^a^
Hours daily sedentary time, Mean (SD)	11.74 (1.19)	11.23 (1.30)	11.35 (1.29)	11.48 (1.28)	11.10 (1.22)	11.25 (1.26)	11.57 (1.31)	11.20 (1.12)	11.39 (1.23)	
Number of daily steps, number (SD)	4575.60 (2247.87)	4839.22 (2168.86)	4777.74 (2186.36)	4701.12 (2774.45)	4996.22 (2342.53)	4877.65 (2527.09)	5688.61 (3073.70)	5452.96 (2593.58)	5571.63 (2844.46)	

a Kruskal–Wallis test.

Participants with higher education levels spent more time in SB (both self-reported and objectively measured) and showed a higher number of daily steps than their counterparts with lower education levels (*p* < 0.000). Regardless of their education level, participants were more sedentary during the week than at weekends (p < 0.000) (Table 1).

Table 2 shows the results of the Kruskal–Wallis test that was used to examine differences between level of education and mentally active or passive SB during weekdays and weekend days. The distribution of mentally active and passive SB was significantly different between levels of education. During weekdays and weekend days, total mentally active SB was associated with the education level (*p* < 0.000).

**Table 2:** Description of mentally active and passive SB time on weekdays and weekend days according to education levels.

	Low primary education	Secondary education	University	*p*
	Mean (SD)	Mean (SD)	Mean (SD)	
Mentally active SB during weekdays (hours/day)				0.000
Computer	0.34 (0.87)	0.42 (0.81)	0.31 (0.70)	
Paperwork	0.6 (0.85)	0.71 (1.01)	1.16 (1.36)	
Reading	0.95 (0.96)	1.26 (1.05)	1.35 (1.12)	
Play instrument	0.02 (0.27)	0.04 (0.25)	0.07 (0.22)	
Artwork	0.54 (1.14)	0.37 (0.89)	0.26 (0.74)	
Total	2.23 (1.96)	2.82 (1.85)	3.17 (2.01)	
Mentally passive SB during weekdays (hours/day)				0.001
TV	3.46 (1.60)	3.33 (1.46)	2.85 (1.53)	
Listening music	0.72 (1.24)	0.63 (1.01)	0.55 (0.84)	
Phone	0.34 (0.36)	0.44 (0.60)	0.42 (0.56)	
Car (co-driver)	0.60 (0.78)	0.81 (0.90)	0.76 (0.68)	
Total	5.13 (2.38)	5.23 (2.22)	4.59 (2.04)	
Mentally active SB during weekends (hours/day)				0.000
Computer	0.36 (0.83)	0.44 (0.91)	0.24 (0.55)	
Paperwork	0.5 (0.84)	0.62 (1.01)	0.82 (0.95)	
Reading	0.98 (1.06)	1.27 (1.06)	1.41 (1.12)	
Play instrument	0.02 (0.18)	0.03 (0.22)	0.07 (0.32)	
Artwork	0.48 (1.11)	0.31 (0.79)	0.20 (0.64)	
Total	2.10 (1.83)	2.55 (1.81)	2.71 (1.75)	
Mentally passive SB during weekends (hours/day)				0.001
TV	3.47 (1.68)	3.37 (1.50)	2.80(1.53)	
Listening music	0.63 (1.12)	0.66 (1.01)	0.59 (0.87)	
Phone	0.33 (0.39)	0.42 (0.53)	0.37 (0.47)	
Car (co-driver)	0.59 (0.66)	0.79 (0.80)	0.82 (0.83)	
Total	5.04 (2.26)	5.25 (2.15)	4.60 (2.02)	

During weekdays, the average of daily hours spent in mentally active SB was 2.23 ± 1.96 in the primary education group, 2.82 ± 1.85 in the secondary education group, and 3.17 ± 2.01 in the university education group. Doing paperwork, reading or playing an instrument was the most common mentally active activities. During weekdays, mentally passive SB (watching television, listening to music, talking on the phone while sitting, or sitting during transport) was higher in participants with primary and secondary education than university education (*p* < 0.001). The mean daily hours spent in mentally passive SB during weekdays were 5.13 ± 2.38 in participants with primary education, 5.23 ± 2.22 in participants with secondary education, and 4.59 ± 2.04 in participants with university-level education (*p* < 0.001) (Table 2).

Table 3 shows the multiple linear regression models with daily SB (measured with SBQ questionnaire and accelerometry) and covariates (level of education, age, gender, comorbidity, and BMI categories). The models explained a relatively small amount of the variability. Education level was associated with self-reported daily SB time (*R*^2^ = 0.026, *p* < 0.000), mentally active SB (*R*^2^ = 0.028, *p* < 0.000), and mentally passive SB (*R*^2^ = 0.034, *p* < 0.000), with higher amounts of self-reported SB time related to lower education levels. However, it was not associated with accelerometer-derived daily SB (*R*^2^ = 0.052, *p* = 0.149). BMI categories were associated with self-reported SB (*R*^2^ = 0.026, *p* < 0.013) and SB accelerometer-derived (*R*^2^ = 0.034, *p* < 0.000), with higher amounts of SB time related to higher BMI.

**Table 3:** Multiple linear regression model adjusted for level of education, age, gender, comorbidities, and BMI categories.

		*Level of education*			Age			*Gender*			*Comorbidity*			*BMI categories*		
	*R* ^2^	Root mean square	*F*	*p*	Root mean square	*F*	*p*	Root mean square	F	*p*	Root mean square	*F*	*p*	Root mean square	*F*	*p*
Total daily SB time with ActiGraph	0.052	2.913	1.909	0.149	42.683	27.973	0.000	38.172	25.017	0.000	1.399	0.917	0.338	5.544	3.633	0.057
SBQ total	0.026	3238.701	8.374	0.000	97.62	0.252	0.615	1898.98	4.910	0.027	152.242	0.394	0.531	4359.11	11.27	0.001
SBQ weekend	0.026	69.290	8.314	0.000	12.451	1.494	0.222	38.647	4.637	0.031	26.092	3.131	0.077	51.317	6.157	0.013
SBQ weekday	0.024	65.069	7.222	0.001	0.319	0.035	0.851	38.798	4.306	0.038	0.180	0.020	0.888	106.901	11.865	0.001
SBQ mentally active	0.028	4.098	16.440	0.000	0.185	0.744	0.389	0.185	0.744	0.389	0.056	0.223	0.637	0.024	0.096	0.757
SBQ mentally passive	0.034	2.755	8.101	0.000	0.039	0.115	0.735	0.583	1.713	0.191	0.271	0.798	0.372	5.553	16.329	0.000

## 4 Discussion

The aim of this cross-sectional study was to examine the relationship between the educational level and self-reported as well as device-measured SB. Our results have shown that education level was significantly associated with self-reported SB but not with total daily SB time assessed with accelerometry. In addition, significant differences were found among mentally active and passive SB. Mentally active SB was higher in highly educated participants, and mentally passive SB was higher in participants with primary and secondary education levels, both during weekdays and weekend days. Gender was associated with overall SB but did not differ between mentally active or passive SB.

To determine the context of sedentary behavior, it is necessary to consider the differences observed in our study between the hours of sedentary behavior measured objectively or subjectively. The subjects who participated in the study were more sedentary than they thought. This fact could be justified through the theory of Parker and Bates in which they conclude that “any difference is information, regardless of whether it makes a difference to someone or something.” On the one hand, the objective part, which in our study would coincide with the sedentary lifestyle measured through the accelerometer, would be considered independent of the observer and the situation. On the other hand, subjective data are considered by the person themselves, and this information makes a difference from someone’s point of view (49, 50). It is easier for participants to be able to remember the time they dedicate to a specific sedentary activity than to remember the total sedentary time (51), although it is important to keep in mind that the questionnaire used is composed of multiple items and tends to underestimate total sedentary time (52). It is important to keep in mind that very active people were not included in the study, and therefore, this could reduce the magnitude of the correlations; this has happened in other studies in which when correlating the objective measures with the subjective ones it is found that these, and they do so weakly (53).

Our results have shown that education level was significantly associated with self-reported SB and was not significantly associated with device-measured total daily SB, similar to the results obtained in other studies (54–58). Our findings suggest that the higher the education level, the lower the self-reported SB time. This could be explained by the fact that people with low levels of education are not as conscious of the negative health impact of prolonged maintenance of SB than the more educated counterparts, such as a higher risk of obesity (59), or increased cardiovascular disease (60) or they may live in environments which encourage sitting and place less value on the benefits of sitting less. The fact that people with lower education levels tend to spend more time in SB should be considered when addressing public health needs (25–27).

In our study, a higher educational level was associated with mentally active SB including activities such as reading, doing paperwork, or playing instruments. In contrast, mentally passive SB activities such as watching television, listening to music, talking on the phone, or traveling in a car as a co-driver were associated with low educational levels. These differences may be due to different causes. For example, people who have attained higher levels of education tend to spend more time in cognitively demanding activities which they find more stimulating than passive activities (61). People with higher levels of education are likely to have more disposable income to spend on leisure time activities, which can positively impact health (62). In addition, they spend less time on mentally passive sedentary activities (58). Our results are like other studies; Van Cauwenberg and Prince concluded that older adults with higher levels of education had a decreased television time, and those with lower education levels had an increased overall mentally passive sedentary time (56, 58). In addition, similar studies reported that higher education level was linked to mentally active SB such as reading (63, 64). In terms of the amount of time adults spend in mentally active and passive SB, Hallgren et al. reported that the average times in mentally active and passive SB time were 193 and 258 min per week, respectively (24).

The current world situation is complex, especially if we focus on the way in which people from previous generations tried to maintain an intellectual and emotional balance. If this imbalance is reached, organic diseases caused by mental illnesses (65) can develop. Therefore, it is important to know the characteristics of older people who tend to be sedentary and be able to influence the reduction of sitting. In our study, knowing that the educational level is related to the level of sitting is an interesting fact that can help develop new health policies focused on adulthood. This future line of work would focus on the responsibility we have as human beings to provide equitable care for the next generations, related to scientific discoveries on health and life protection (65). In this case, the health and educational systems must join forces to reinforce the personal and collective security of individuals (65).

There is an association between gender and sedentary behaviors, and it varies throughout life. In each of the age groups, there is an association between certain sedentary activities and gender. In our study, gender was associated with device-measured and subjective measures of SB. Device-measured SB was higher in men with primary education levels than with higher levels of education. In contrast, self-reported SB was higher in women with all levels of education and increased with the level of education. To date, there are studies that present results similar to ours in which it is concluded that gender was associated with SB (66, 67). However, there are other scientific studies carried out in the older adult population in which men are more sedentary than women, analyzing it from the objective and subjective perspective (68, 69).

One of the strengths of this study was to analyze the association between educational level and sedentary time measured objectively and subjectively in a cohort of community-dwelling older adults.

from four European countries. In addition, it also studies the difference between sedentary time, depending on whether the activity performed is mentally active or passive. This study also has limitations. First, the accelerometer had some well-known limitations for assessing posture that could be overcome using an inclinometer (e.g., time spent standing is likely to be classified as sedentary using an accelerometer). Second, by excluding active people from this study, it is possible that the correlation presented is affected, and furthermore, the data obtained would only be extrapolated to insufficiently active older people.

## 5 Conclusion

Findings from this study reveal that there is a significant association between the education level with subjective SB in European older adults but not for device-measured SB. In the decade of healthy aging (2020–2030) (WHO), a worldwide situation in which life expectancy is increasing, but living longer does not go hand in hand with living healthier, identifying people at higher risk of poorer health outcomes is relevant and compelling. Having low education levels has shown to be an indicator of health inequalities such as higher sedentary time, which, in turn, may be related to decreased functional performance and risk of dependency in the longer term. There is a need to identify the most vulnerable groups and target them as a priority for health-based interventions. There is a need to enhance higher education levels among the population, focus on those with lower education levels, and design preventive strategies to decrease overall SB (and enhance movement), and more specifically mentally passive sedentary activities.

## Scope statement

This study is a step forward toward the knowledge of the association between the education level of older adult people and sedentary behavior. In addition, we would like to study the association between the mentally active or passive activities and level of education. These results are significantly important since low education level in older adults is associated with self-reported sedentary but not with an objective sedentary measure. These results are important to identify the groups of older adults who need specifically interventions to decrease sedentary behavior. This study is based on the major problem of older adult people, sedentary behavior.

That means the readers of this journal will definitely be interested in reading this study as it is a novel topic, and it can be used for improving the knowledge about it.

## Data availability statement

The original contributions presented in the study are included in the article/Supplementary material, further inquiries can be directed to the corresponding author.

## Author contributions

BR-R: Writing – original draft, Writing – review & editing. MT: Writing – original draft, Writing – review & editing. OS-N: Writing – original draft, Writing – review & editing. PC: Writing – original draft, Writing – review & editing. LC-P: Writing – original draft, Writing – review & editing. MR: Writing – original draft, Writing – review & editing. JB: Writing – original draft, Writing – review & editing. NB: Writing – original draft, Writing – review & editing. JW: Writing – original draft, Writing – review & editing. DR: Writing – original draft, Writing – review & editing. EM: Writing – original draft, Writing – review & editing. MD: Writing – original draft, Writing – review & editing. EA-G: Writing – original draft, Writing – review & editing. MG-G: Writing – original draft, Writing – review & editing.
