# Supplementary material for: Is education level, as a proxy for socio-economic position, related to device-measured and self-reported sedentary behavior in European older adults? A cross-sectional study from the SITLESS project
Source: Front Public Health. 2023 Dec 19;11:1296821. doi: 10.3389/fpubh.2023.1296821 (PMC10758416; doi:10.3389/fpubh.2023.1296821)
Supplement: Supplementary file 1 [file Table_1.DOCX]

Appendix

Table 1: Characteristics of the overall sample according to the education level and divided by gender

| Education |  | Primary |  | Secondary |  |  | University |  |  |  |
| --- | --- | --- | --- | --- | --- | --- | --- | --- | --- | --- |
|  | *Male (n=66)* | Female (n=217) | Total (n=283) | Male (n=266) | Female (n=396) | Total (n=662) | Male (n=140) | Female (n=138) | Total (n=278) |  |
| BMI, number (%): |  |  |  |  |  |  |  |  |  | 0.151a |
| Underweight | - | - | - | - | 4 (1) | 4 (0.6) | - | 1 (0.7) | 1 (0.4) |  |
| Normal weight | 6 (9.1) | 47 (21.7) | 53 (18.7) | 43 (16.2) | 106 (26.8) | 149 (22.5) | 33 (23.6) | 39 (28.2) | 72 (25.9) |  |
| Overweight | 36 (54.5) | 79 (36.4) | 115 (40.6) | 131 (49.2) | 138 (35.1) | 270 (40.8) | 71 (50.7) | 50 (36.2) | 121 (43.5) |  |
| Obese | 24 (36.4) | 91 (41.9) | 115 (40.6) | 92 (34.6) | 147 (37.1) | 239 (36.1) | 36 (25.7) | 48 (34.8) | 84 (30.2) |  |
| Country, number (%): |  |  |  |  |  |  |  |  |  | 0.000a |
| Spain | 30 (45.5) | 128 (59) | 158 (55.8) | 24 (9) | 67 (16.9) | 91 (13.7) | 9 (6.4) | 24 (17.4) | 33 (11.9) |  |
| Germany | 4 (6.1) | 6 (2.8) | 10 (3.5) | 78 (29.3) | 145 (36.6) | 223 (33.7) | 52 (37.1) | 24 (17.4) | 76 (27.3) |  |
| United Kingdom | 3 (4.5) | 16 (7.4) | 19 (6.7) | 74 (27.8) | 86 (21.7) | 160 (24.2) | 62 (44.3) | 67 (48.6) | 129 (46.4) |  |
| Denmark | 29 (43.9) | 67 (30.9) | 96 (33.9) | 90 (33.8) | 98 (24.7) | 188 (28.4) | 17 (12.1) | 23 (16.7) | 40 (14.4) |  |
| Number medical conditions, | 3 (9) | 3 (10) | 3 (10) | 2 (10) | 3 (16) | 3 (16) | 2 (8) | 2 (10) | 2 (10) | <0.001b |
| median (range) |  |  |  |  |  |  |  |  |  |  |
|  |  |  |  |  |  |  |  |  |  |  |

Table 2: Characteristics of the overall sample according to the country

|  | Spain (n=282) | Germany (n=309) | United Kingdom(n=308) | Denmark (n=295) | overall sample (n=1223) |
| --- | --- | --- | --- | --- | --- |
|  |  |  |  |  |  |
| Age (years), mean (SD) | 75.93 (6.42) | 74.88 (6.18) | 72.59 (5.52) | 77.32 (5.66) | 75.20 (6.19) |
| Gender, number (%): |  |  |  |  |  |
| Male | 63 (22.3) | 134 (43.4) | 139 (45.1) | 107 (36.3) | 472 (38.6) |
| Female | 219 (77.7) | 175 (56.6) | 169 (54.9) | 188 (63.7) | 751 (61.4) |
| BMI categories, number (%): |  |  |  |  |  |
| Underweight | - | 2 (0.6) | 1 (0.3) | 2 (0.7) | 5 (0.4) |
| Normal weight | 41 (14.5) | 59 (19.1) | 66 (21.4) | 104 (35.2) | 274 (22.4) |
| Overweight | 112 (39.7) | 136 (44) | 127 (41.2) | 115 (39) | 506 (41.4) |
| Obese | 129 (45.7) | 112 (36.2) | 114 (37) | 74 (25.1) | 438 (35.8) |
| Number of medical conditions, | 3 (9) | 3 (9) | 2 (8) | 3 (8) | 2 (10) |
| median (range) |  |  |  |  |  |
| Educational level, number (%): |  |  |  |  |  |
| Primary | 158 (56) | 10 (3.2) | 19 (6.2) | 67 (22.7) | 283 (23.1) |
| Secondary | 91 (32.3) | 223 (72.2) | 160 (51.9) | 188 (63.7) | 662 (54.1) |
| University | 33 (11.7) | 76 (24.6) | 129 (41.9) | 40 (13.6) | 278 (22.7) |
| SBQ hours/day, mean (SD) | 7.47 (3.34) | 7.69 (2.45) | 7.91 (2.85) | 8.03 (2.72) | 7.75 (2.85) |
| SBS WD hours/day, mean (SD) | 7.61 (3.66) | 7.72 (2.56) | 8.14 (3.12) | 7.94 (2.77) | 7.83 (3.04) |
| SBQ WE hours/day, mean (SD) | 7.12 (23.39) | 7.58 (2.64) | 7.31 (2.82) | 8.23 (2.89) | 7.54 (2.91) |
| Accelerometry: |  |  |  |  |  |
| Hours daily sedentary time, | 11.28 (1.29) | 11.21 (1.33) | 10.95 (1.14) | 11.76 (1.14) | 11.31 (1.26) |
| mean (SD) |  |  |  |  |  |
| Number daily steps, number (SD) | 5139.56 (2261.23) | 4425.02 (2416.09) | 5791.36 (2841.56) | 4735.32 (2415.39) | 5012.28 (2546.62) |
